# The Innate Immune DNA Sensing cGAS-STING Signaling Pathway Mediates Anti-PRRSV Function

**DOI:** 10.3390/v13091829

**Published:** 2021-09-14

**Authors:** Yulin Xu, Youwen Zhang, Shaohua Sun, Jia Luo, Sen Jiang, Jiajia Zhang, Xueliang Liu, Qi Shao, Qi Cao, Wanglong Zheng, Nanhua Chen, François Meurens, Jianzhong Zhu

**Affiliations:** 1 College Veterinary Medicine, Yangzhou University, Yangzhou 225009, China; ylxu15650096726@163.com (Y.X.); zyw18252734747@163.com (Y.Z.); shaohuas90@sina.com (S.S.); luojiajia20210518@163.com (J.L.); jiangsen8888888@163.com (S.J.); awaw0601@163.com (J.Z.); xueliangaaa@foxmail.com (X.L.); sq970123@163.com (Q.S.); qc_2021@163.com (Q.C.); 007297@yzu.edu.cn (W.Z.); hnchen@yzu.edu.cn (N.C.); 2 Joint International Research Laboratory of Agriculture and Agri-Product Safety, Yangzhou University, Yangzhou 225009, China; 3 Comparative Medicine Research Institute, Yangzhou University, Yangzhou 225009, China; 4 Jiangsu Co-Innovation Center for Prevention and Control of Important Animal Infectious Diseases and Zoonoses Yangzhou, Yangzhou 225009, China; 5 BIOEPAR, INRAE, Oniris, 44307 Nantes, France; francois.meurens@inra.fr; 6 Department of Veterinary Microbiology and Immunology, Western College of Veterinary Medicine, University of Saskatchewan, Saskatoon, SK S7N 5E2, Canada

**Keywords:** PRRSV, innate immunity, DNA sensor, cGAS-STING, antiviral activity

## Abstract

Porcine reproductive and respiratory syndrome virus (PRRSV) modulates host innate immunity which plays a key role against PRRSV infection. As a RNA virus, PRRSV is mainly sensed by innate immune RNA receptors, whereas the role of innate immune DNA sensors in the PRRSV infection has not been elucidated. Here, we investigated the roles of DNA sensing cGAS-STING pathway in both PRRSV infected Marc-145 cells and porcine macrophages. The results show that in Marc-145 cells, the stable expression of STING with or without stimulations exhibited anti-PRRSV activity, and STING knockout heightened PRRSV infection. In CD163-3D4/21 porcine macrophages, either expression of STING or stimulation of cGAS-STING signaling obviously suppressed PRRSV infection, whereas in STING knockdown macrophages, the PRRSV infection was upregulated. Our results clearly demonstrate that the host cGAS-STING signal exerts an important antiviral role in PRRSV infection.

## 1. Introduction

Porcine reproductive and respiratory syndrome (PRRS) has emerged and evolved for almost three decades, representing one of the most widespread and important swine diseases [1]. It is characterized by respiratory symptoms in piglets and abortion, reproductive failure, fetal death, and congenital infections in pregnant sows [2]. The porcine reproductive and respiratory syndrome virus (PRRSV) belongs to the genus *betaarterivirus*, family *Arteriviridae* in the order *Nidovirales* [3], has a genome of positive-sense single-stranded RNA of 15.4 kb in length with the 5′-cap and 3′-polyadenylation. The PRRSV genome contains 11 open reading frames (ORFs), encoding 16 nonstructural proteins and 8 structural proteins [4]. The ORF1 at the 5′ end of the viral genome encodes 16 nonstructural proteins which are nsp1α, nsp1β, nsp2, nsp2TF, nsp2N, nsp3-6, nsp7α, nsp7β, and nsp8-12, whereas ORF2-ORF7 at the 3′ end of the viral genome encodes eight viral structural proteins including GP2a, GP3, GP4, GP5, E, ORF5a, M and N. PRRSV is grouped into two species PRRSV1 and PRRSV2 based on the genomic genetic differences [5]. 

The innate immune system acts as the first line of defense against pathogen infection. Pathogen-associated molecular patterns (PAMPs) during infection are recognized by host germline-encoded pattern recognition receptors (PRRs), including Toll-like receptors (TLRs), RIG-I-like receptors (RLRs), NOD-like receptors (NLRs), C-type lectin receptors (CLRs), and cytosolic DNA receptors (CDRs) [6,7,8]. The signaling pathways associated with these PRRs trigger either gene transcription induction or gene transcription independent protease activation, which together orchestrate the anti-infection response. To fight against viruses, host innate immunity induces antiviral type I interferon (IFN) transcription and production, mainly via TLR, RLR and CDR elicited signaling cascades [9]. As a RNA virus, PRRSV has been thought to be recognized by TLR3/7/8/9 and RLRs, which trigger innate antiviral type I IFN signaling [10].

PRRSV has coevolved with host in an intricate and delicate way. On the one hand, PRRSV has evolved a number of strategies to modulate host immune responses, especially antiviral type I IFNs in swine [11]. Specifically, six PRRSV proteins including nsp1α, nsp1β, nsp2, nsp4, nsp11, and N have been shown to suppress IFN responses [12,13,14]. On the other hand, type I IFNs (IFNα/β) as the most potent component of innate immunity fighting against viruses are effective against PRRSV [15]. Multiple host factors that are IFN stimulated genes (ISGs) have been identified to be host restriction factors against PRRSV, including interferon-induced protein with tetratricopeptide repeats 3 (IFIT3) [16], guanylate-binding protein 1 (GBP1) [17], myxovirus resistance protein 1 (Mx1) [18], zinc finger antiviral protein (ZAP) [19], Viperin [20], interferon gamma-inducible protein 16 (IFI16) [21], cholesterol 25-hydroxylase (CH25H) [22], Tetherin and interferon-induced transmembrane protein 1 (IFITM1) [23] and tripartite motif-containing 25 (TRIM25) [24].

The cGAS-STING has been defined as the most critical members of CDRs, which plays a key role in sensing DNA virus infection [25]. The DNA sensor cGAS is a cyclic GMP-AMP (cGAMP) synthase, and catalyzes the synthesis of 2′3′-cGAMP from ATP and GTP. Then, 2′3′-cGAMP, as a second message, activates the signaling adaptor STING to induce antiviral IFN response. However, accumulated evidence shows that cGAS-STING pathway harbors a broad range antiviral function [26,27]. The DNA pathway during PPRSV infection has not been investigated and its role remains unclear. In this study, we evaluated the anti-PRRSV activity by cGAS-STING pathway in both Marc-145 cells and porcine macrophages. 

## 2. Materials and Methods

### 2.1. Cells, Virus and Reagents

PRRSV-permissive Marc-145 cells were cultured in Dulbecco’s Modified Eagle’s Medium (DMEM; Life Technologies Corp., Grand Island, NY, USA) supplemented with 10% fetal bovine serum (FBS) and 100 U/mL of penicillin plus 100 μg/mL streptomycin. Porcine alveolar macrophages (3D4/21, ATCC CRL-2843) were cultured in RPMI (Hyclone Laboratories, Logan, UT, USA) containing 10% FBS with penicillin/streptomycin. All cells were maintained at 37 °C with 5% CO_2_ in a humidified incubator. The PRRSV strain used in this study was highly pathogenic (HP) PRRSV XJ17-5 (GenBank ID: MK759853.1), which was isolated from Xinjiang Province, China, being the representative strain of HP-PRRSV2 predominant in Chinese swine herds [28]. The virus was propagated and titrated in Marc-145 cells grown in DMEM supplemented with 2% FBS. G418 and puromycin were purchased from Sigma (Shanghai, China). Gateway^®^ LR Clonase^TM^ II Enzyme mix were from ThermoFisher Scientific (Waltham, MA, USA) polydA:dT and 2′3′-cGAMP were bought from InvivoGen (Hong Kong, China).

### 2.2. Establishment of Marc-145 Cell Line Stably Expressing Porcine STING

Marc-145 cells were transfected with the plasmid pEGFP-pSTING or vector control pEGFP-C1 using the Lipofectamine 3000 reagent (ThermoFisher Scientific) according to the manufacturer’s protocol. GFP-STING and vector GFP expressing cells were both selected with 800 μg/mL of G418 diluted in DMEM containing 10% FBS. The medium containing G418 was replenished every 3–4 days during selection. After selection, individual cell clones were isolated by a limited dilution method using 96-well cell culture plates, and the cell clones expressing the GFP-STING and vector GFP were chosen and designated as Marc-145-GFP-STING and control Marc-145-GFP, respectively.

### 2.3. Construction of Recombinant HP-PRRSV XJ17-5 Strain Carrying dsRed

To generate an infectious clone carrying the dsRed encoding sequence, a full-genome cDNA clone of the HP-PRRSV XJ17-5 isolate was firstly constructed as we previously described [29]. Briefly, three overlapping segments (F1, F2 and F3) spanning the full-length genome of XJ17-5 were generated by PCR amplification using the high fidelity Primestar Max DNA polymerase (TAKARA, Beijing, China) and a set of primers (Table 1). The full-length XJ17-5 cDNA clone pACYC177-CMV-rXJ17-5 (rXJ17-5) was obtained by sequential cloning of three segments using 4 unique restriction enzyme sites (*Pac*I, *Afl*II, *Asc*I and *Not*I). The dsRed was PCR amplified from pDsRed-C1 using the primers shown in Table 1. The dsRed PCR product was double enzyme digested and ligated into the *Kpn*I and *Bcl*I sites of F3 segment of pACYC177-XJ17-5-F3-EGFP prepared in our separate study, where the dsRed gene coding is to replace EGFP coding located between the non-structural and structural genes. Then, the generated new F3 segment was cut off by *Asc*I and *Not*I from recombinant pACYC177-CMV-XJ17-5-F3, and ligated into the same sites of pACYC177-CMV XJ17-5-F1-F2 to obtain the full length of pACYC177-CMV-XJ17-5 (rXJ17-5-dsRed) [29,30]. The obtained full-length rXJ17-5-dsRed clone was transfected into BHK-21 cells using Lipofectamine 3000 reagent and cell culture supernatants obtained at 48 h post transfection were serially passaged on Marc-145 cells to rescue the PRRSV rXJ175-5-dsRed. The real-time infection of rXJ17-5-dsRed virus can be visualized by fluorescence microscopy.

### 2.4. CRISPR gRNA Design and Preparation of STING^−/−^ Marc-145 Cells

The CRISPR gRNAs targeting monkey STING (GenBank accession: NC_041759.1) were designed using the web tool from Benchling (www.benchling.com, accessed on 9 September 2021). Two gRNA 1 and gRNA 2 were chosen according to the predicted scores, and the encoded DNA sequences are shown in Table 2. The annealed gRNA encoding DNA pairs were ligated with the *Bbs*I digested vector pSpCas9(BB)-2A-GFP (pX458, Addgene, Watertown, NY, USA). Subsequently, gRNA expressing pX458 was transfected into Marc-145 cells using Lipofectamine 3000 reagent and the GFP-positive cells were sorted out from transfected cells using a FACS Aria SORP cell sorter (Becton Dickinson). The individual Marc-145 cell clones obtained by limited dilution method from the sorted GFP expressing cells were screened by PCR using the designed primers shown in Table 2. The PCR products were cloned into T vector using pClone007 Versatile Simple Vector Kit (TsingKe Biological Technology, Beijing, China), the inserted fragments were multiply sequenced and the sequences were analyzed for base insertion and deletion (indel) mutations, based on which, four STING^−/−^ and one STING^+/−^ Marc-145 cell clones were obtained (Supplementary Figure S1 and Figure 3A).

### 2.5. Porcine CD163 Gene Cloning and Establishment of Porcine Macrophages Stably Expressing CD163 for PRRSV Infection

The coding region of the pCD163-EGFP fusion protein was PCR amplified from pEGFP-N1-pCD163 preserved in our lab, using the primer pairs: Forward-*Sal*I: 5′-GGGCC*GTCGAC*ATGGTGCTACTTGAAGACTCTGGATCTG-3′ and Reverse-*EcoR*V: 5′-GGCCC*GATATC*TTACTTGTACAGCTCGTCCATGCCG-3′. The PCR product was digested with *Sal*I/*EcoR*V and cloned into pENTR4 vector (Addgene). The sequence confirmed pCD163-EGFP were transferred from pENTR4 vector to lentiviral vector pLenti CMV Puro DEST (Addgene) by LR recombination. The CD163-EGFP expressing lentiviruses were generated by co-transfecting pCD163-EGFP lentiviral vector with package plasmids psPAX2 and pMD2.G into 293T cells using Lipofectamine 2000 (ThermoFisher Scientific). The supernatant containing pCD163-EGFP expressing lentiviruses were used to infect the porcine alveolar macrophages (3D4/21, PAMs), and the infected cells were selected with 2 μg/mL puromycin, by replacing with new medium every 3–4 days. About 2 weeks later, individual cell clones were screened for GFP expression and supporting PRRSV replication. The desired cell clone was used for subsequent PRRSV infection experiments.

### 2.6. Porcine STING RNA Interference by siRNA

All siRNAs targeting porcine STING (mRNA accession number: NM_001142838.1) were designed and synthesized by Invitrogen (ThermoFisher Scientific) and the siRNA sequences are listed in Table 3. CD163-PAMs were plated into 24-well plates and the cells with 70–80% confluency were transfected with 30–50 nM of siRNA using Lipofectamine 2000 according to the manufacturer’s instructions. To determine the efficiency of the knockdown, total RNA was extracted from the cells at 24 h post transfection, and endogenous mRNA levels of STING were quantified by RT-qPCR. In parallel, cells were lysed, and proteins were measured by Western blotting for STING protein expression with polyclonal anti-STING rabbit antibody (1:2000) (Proteintech, Rosemont, IL, USA).

### 2.7. Quantitative Reverse Transcription Polymerase Chain Reaction (RT-qPCR)

PRRSV infected Marc-145 or CD163-PAMs were washed three times with PBS, and the total RNAs were extracted using TRIzol reagent (Invitrogen, ThermoFisher Scientific). The extracted RNA was reverse transcribed into cDNA with HiScript^®^ 1st Strand cDNA Synthesis Kit (Vazyme, Nanjing, China) according to the manufacturer’s instructions. Quantitative PCR (qPCR) reaction (20 μL) was performed on a StepOne Plus real-time PCR system (Applied Biosystems, Foster City, CA, USA) using ChamQ Universal SYBR qPCR Master Mix (Vazyme, Nanjing, China) and regular qPCR amplification program. The gene primers used for qPCR amplification are listed in Table 4 and cellular β-actin mRNA was measured as an internal reference control. Relative mRNA expression was calculated using the 2^−ΔΔCt^ method.

### 2.8. Western Blot Analysis

Cells were collected and lysed in radio-immunoprecipitation assay (RIPA) buffer (50 mM Tris pH 7.2, 150 mM NaCl, 1% sodium deoxycholate, 1% Triton X-100). The cleared cell lysates were separated by 15% SDS-PAGE and proteins were transferred onto polyvinylidene difluoride (PVDF) membranes. After being blocked with 5% skim milk for 1 h at room temperature, the membrane was incubated with either mouse anti-PRRSV N protein (Npro) antiserum (1:1000) or other primary antibodies. Next, the membrane was incubated with HRP-conjugated goat anti-mouse or rabbit IgG (1:10,000, Transgen Biotech, Beijing, China) after TBST washing. The protein signal was visualized by Western blot imaging system (Tanon, Shanghai, China) using an ECL chemiluminescent detection system (Tanon, Shanghai, China) according to the manufacturer’s instructions.

### 2.9. Flow Cytometry

Marc-145 or CD163-PAMs were transfected or stimulated with agonists, and then infected with PRRSV XJ17-5-dsRed. At different time points post infection, cells were collected and analyzed by FACSVerse flow cytometer (BD Biosciences, Franklin Lakes, NJ, USA) for GFP and dsRed expressions. All samples were gated based on forward scatter (FSC) and side scatter (SSC) to gate out cellular debris or dead cells. The levels of GFP and dsRed were detected using the wavelength pairs 488/525 nm and 510/600 nm, respectively. The final analysis and graphical output were performed using FlowJo software (Tree Star, Inc., Ashland, OR, USA).

### 2.10. Virus TCID50 Titration

Marc-145 cells grown in 96-well plates were infected with ten-fold serial dilutions of PRRSV samples. After 1 h incubation at 37 °C, the supernatants were replaced with fresh DMEM containing 2% FBS. Five days post infection, the cytopathic effect (CPE) characterized by clumping and shrinkage of cells was obviously visible in Marc-145 cells and the viral titers, expressed as 50% tissue culture infective dose (TCID50), were calculated according to the method of Reed-Muench [31].

### 2.11. Statistical Analysis 

The data are presented as the means ± standard deviations (SD, *n* = 3). Statistical significance between groups was determined by performing a Student *t* test with GraphPad Prism 6.0 software. The *p* value of <0.05 was considered statistically significant.

## 3. Results

### 3.1. Stable Expression of Porcine STING Suppresses PRRSV Infection in Marc-145 Cells

**Figure 1:**
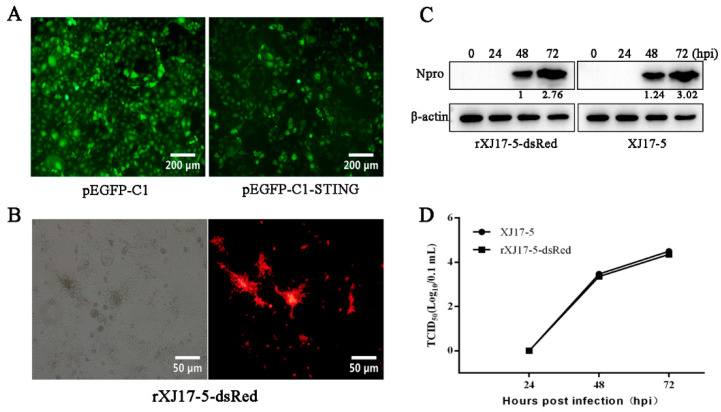
Porcine STING stable Marc-145 cells and characterization of the rescued rXJ17-5-dsRed PRRSV. (**A**) Porcine STING stable Marc-145 cells (right) and vector pEGFP-C1 control stable Marc-145 cells (left) obtained by G418 selection. (**B**) Marc-145 cells were infected with rXJ17-5-dsRed PRRSV at a MOI of 0.1, with cytopathic effect (CPE) and dsRed expression at 48 h post infection (hpi). (**C**,**D**) The in vitro growth kinetics of the cloned rXJ17-5-dsRed virus and the parental XJ17-5 virus. Marc-145 cells were infected with PRRSV at a MOI of 0.1. The growth curve of both PRRSVs in Marc-145 cells within 72 hpi were determined by Western blotting (**C**) and TCID50 assay (**D**). The values of PRRSV N protein (Npro) quantification by gray scanning were shown below the bands following normalization by β-actin.

To explore the role of STING in PRRSV infection, we first prepared the porcine STING stable expressing Marc-145 cells and vector (pEGFP-C1) control stable Marc-145 cells, both of which have GFP signal maintained after G418 selection (Figure 1A). To quantify the PRRSV infection by flow cytometry, we also constructed the recombinant PRRSV carrying the dsRed signal (rXJ17-5-dsRed) (Figure 1B). The rPRRSV-dsRed exhibited similar growth kinetics to parent PRRSV (rXJ17-5) in both Western blotting (Figure 1C) and TCID50 assay (Figure 1D). 

**Figure 2:**
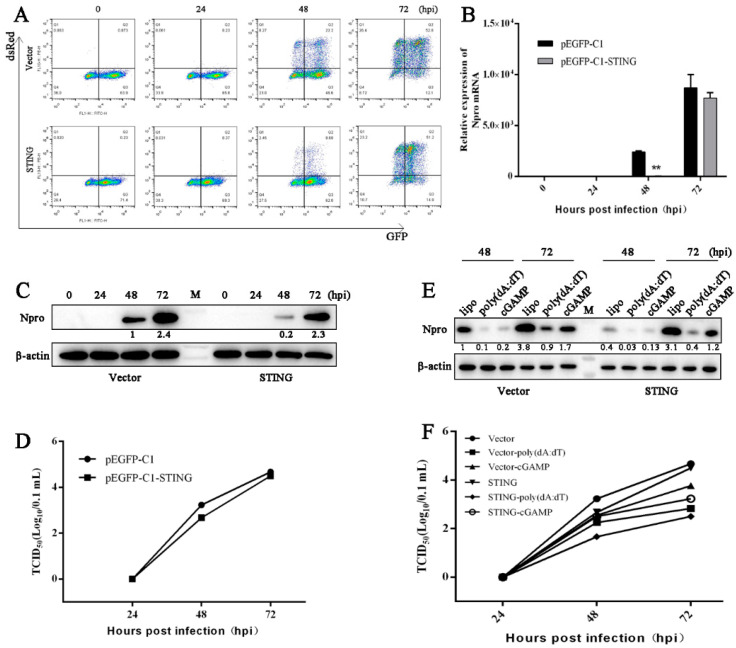
The replication of PRRSV in porcine STING stable Marc-145 cells with or without stimulations. (**A**–**D**) The STING or vector control stable Marc-145 cells seeded in 24-well plates were infected with rXJ17-5-dsRed at a MOI of 0.1, for 0, 24, 48, 72 h, and then assayed by flow cytometry (**A**), RT-qPCR (**B**), Western blotting (**C**) and TCID50 (**D**). The dsRed/GFP double signal represents that those Marc-145 cells stably expressing STING or control GFP were infected by rXJ17-5-dsRed. (**E**,**F**) Marc-145 cells stably expressing STING or control GFP grown in 24-well plates were stimulated by transfection of poly (dA:dT) (1 μg/mL) or 2′3′-cGAMP (1 μg/mL) for 24 h using transfection reagent lipofectamine 2000 (lipo). Then, the cells were infected with PRRSV at a MOI of 0.1, for 24, 48, and 72 hpi, respectively. The infected cells were assayed by Western blotting (**E**) and TCID50 (**F**). ** *p* < 0.01 versus the vector pEGFP-C1 control groups.

The rPRRSV-dsRed infection of GFP-STING and GFP control stable Marc-145 cells were quantified by flow cytometry, and the results show that either dsRed signal or dsRed/GFP double signals were decreased in GFP-STING cells relative to GFP vector control cells, in particular at 48 h post infection (hpi), indicating the antiviral role of STING (Figure 2A). Similar trends were obtained in RT-qPCR analysis of N mRNA (Figure 2B) and Western blotting assay of N protein (Figure 2C), as well as virus titer analysis by TCID50 assay (Figure 2D). 

Further, the stable Marc-145 cells were stimulated with poly (dA:dT) and 2′3′-cGAMP to activate cGAS-STING and STING, respectively, before PRRSV infection. Both agonists inhibited the PRRSV replication as evidenced by Western-blotting (Figure 2E) and TCID50 assay (Figure 2F). In addition, the more pronounced decreases of PRRSV replication in GFP-STING Marc-145 cells were observed relative to control vector GFP Marc-145 cells (Figure 2E,F). Essentially, the results are consistent with those in STING transiently transfected Marc-145 cells we considered in another separate study. 

### 3.2. The Endogenous STING in Marc-145 Cells Mediates Antiviral Activity against PRRSV

**Figure 3:**
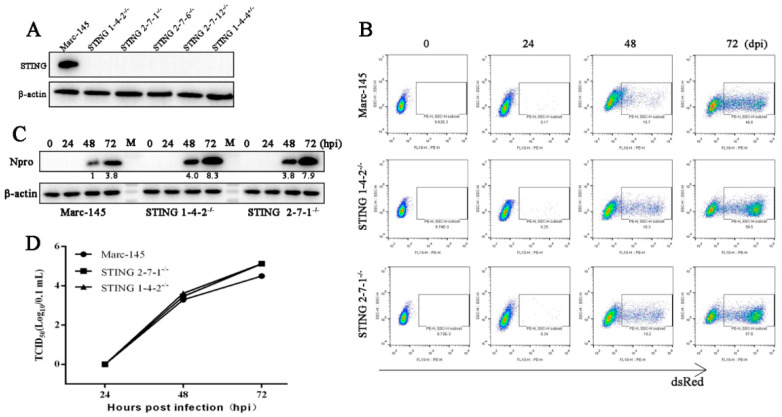
The replication of PRRSV in STING^−/−^ Marc-145 cells. (**A**) Detection of STING protein expression in 4 STING^−/−^ Marc-145 cell clones and 1 STING^+/−^ Marc-145 cell clone by Western blotting. (**B**–**D**) Two STING^−/−^ cell clones and control Marc-145 cells in 24-well plates were infected with rXJ17-5-dsRed at a MOI of 0.1. Cells were harvested at 0, 24, 48, and 72 hpi, and analyzed by flow cytometry. The dsRed signal represents the Marc-145 cells infected by PRRSV (**B**). The expression of PRRSV N proteins (Npro) was detected by Western blotting (**C**). Viral titers in the supernatants were assayed by TCID50 (**D**).

Previously, we were able to observe the upregulated PRRSV replication in STING siRNA treated Marc-145 cells in another study. Here, we further made the homologous STING knockout Marc-145 cell clones by CRISPR-Cas9 method. Four homologous STING knockout cell clones were obtained and two cell clones (2-7-1 and 1-4-2) were selected for subsequent experiments (Figure 3A and Supplementary Figure S1). Compared with Marc-145 cells, the PRRSV replications were heightened at 48, 72 h post infection in both STING knockout cell clones as evidenced by flow cytometry (Figure 3B), Western blotting (Figure 3C) and TCID50 assay (Figure 3D). Taken together, these results suggested that STING exerts antiviral activity against PRRSV in Marc-145 cells.

### 3.3. Both Ectopic STING and Endogenous STING Inhibit PRRSV Replication in Porcine Macrophages

**Figure 4:**
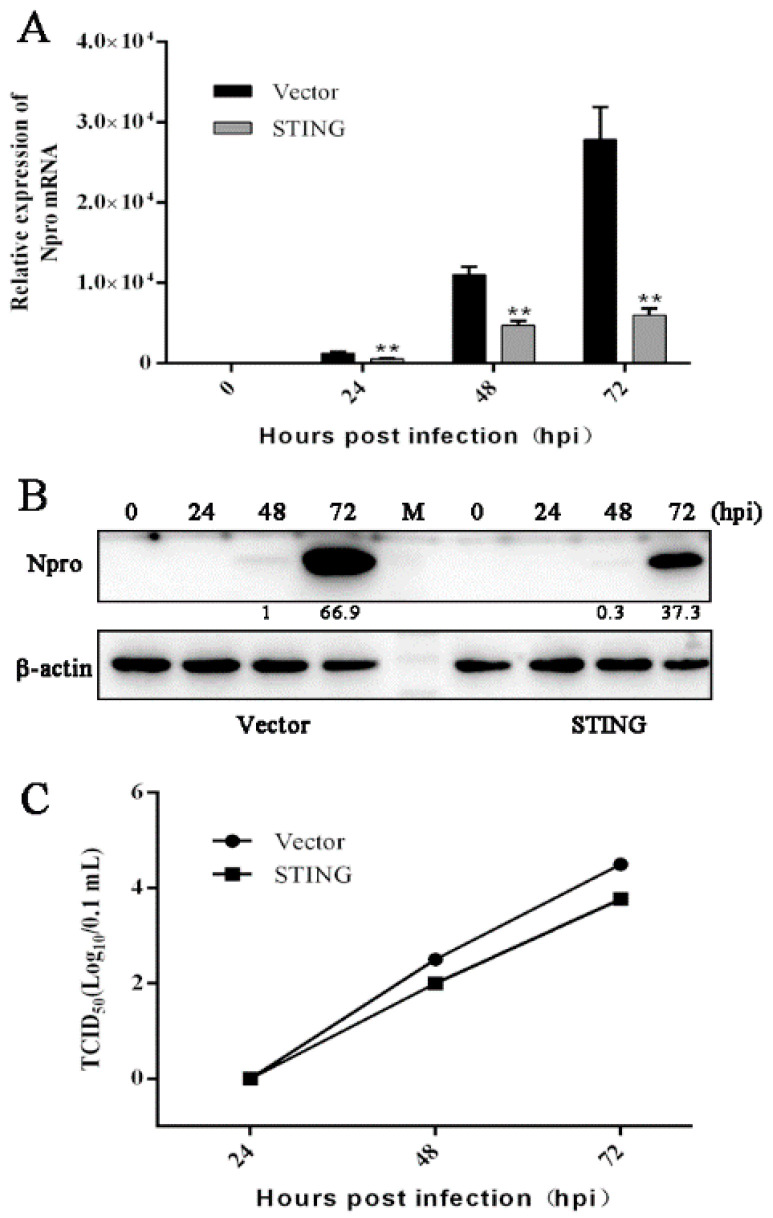
The PRRSV replication in porcine macrophages transfected with porcine STING. (**A**–**C**) CD163-PAMs seeded in 24-well plates were transfected with porcine STING or vector pmCherry-C1 (0.25 μg each) using lipofectamine 2000. At 24 h post transfection, the cells were infected with PRRSV at a MOI of 0.1, for 0, 24, 48, and 72 h, respectively. The infected cells were then assayed by RT-qPCR (**A**), Western blotting (**B**) and TCID50 assay (**C**). ** *p* < 0.01 versus the vector control groups. The values of PRRSV N protein (Npro) quantification by gray scanning were shown below the bands following normalization by β-actin.

To investigate the anti-PRRSV role of STING in porcine macrophages, we developed CD163-PAMs (3D4/21), which stably express GFP tagged porcine CD163 and successfully support PRRSV replication. First, the CD163-PAMs were transfected with porcine STING and infected with PRRSV. The results from the experiment show that ectopic STING suppressed the N gene transcription in RT-qPCR (Figure 4A), N protein expression in Western blotting (Figure 4B) and virus particle secretion in TCID50 assay (Figure 4C).

**Figure 5:**
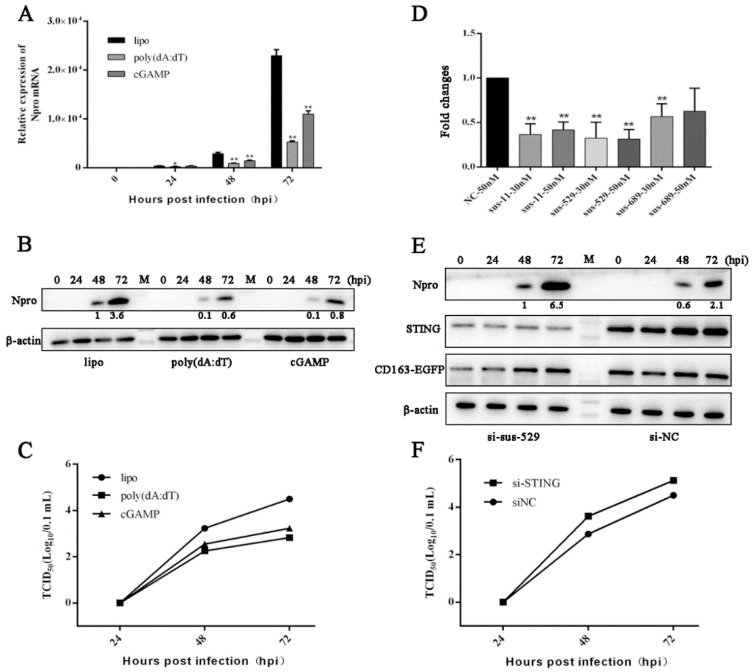
The PRRSV replication in porcine macrophages treated with agonists for cGAS-STING or with STING siRNA. (**A**–**C**) CD163-PAMs grown in 24-well plates were stimulated by transfection of poly (dA:dT) (1 μg/mL) or 2′3′-cGAMP (1 μg/mL) using lipfectamine 2000 (lipo) for 24 h. Next, the stimulated cells were infected with PRRSV at a MOI of 0.1, for 0, 24, 48, and 72 h, respectively. The infected cells were assayed by RT-qPCR (**A**), Western blotting (**B**) and TCID50 (**C**). (**D**–**F**) CD163-PAMs in 24-well plates were transfected with the indicated siSTING or siNC by lipofectamine 2000 for 48 h, and STING gene expressions were quantified by RT-qPCR (D). CD163-PAMs were treated by transfection with siNC, and siSTING-529, respectively, for 48 h, followed by infection with PRRSV at a MOI of 0.1. Cells were harvested at 0, 24, 48, and 72 hpi, and the indicated proteins were detected by Western blotting (**E**). Viral titers in the supernatants were assayed by TCID50 (**F**). * *p* < 0.05, ** *p* < 0.01 versus the lipo/siNC control groups.

Second, the CD163-PAMs were stimulated with poly (dA:dT) and 2′3′-cGAMP to activate cGAS-STING and STING, respectively, before PRRSV infection. The results show that both agonists suppressed the PRRSV infection, at 48 and 72 hpi, as evidenced by N gene transcription in RT-qPCR (Figure 5A), N protein expression in Western blotting (Figure 5B), and virus titers in TCID50 assay (Figure 5C).

Third, the CD163-PAMs were treated with STING siRNA to knockdown STING for subsequent PRRSV infection. To this end, three pairs of STING siRNAs were tested for the knockdown efficiency by RT-qPCR, and the most effective siRNA 529 was selected for the next experiments (Figure 5D). In STING siRNA treated CD163-PAMs, the STING protein expressions were obviously declined as expected, relative to negative siRNA (siNC) treated cells, whereas the GFP-CD163 were comparable (Figure 5E). Simultaneously, the viral N proteins were substantially increased in STING knockdown cells (Figure 5E), and the virus titers in STING knockdown cells were also upregulated (Figure 5F). Taken together, the results demonstrate clearly that STING exerts anti-PRRSV in both Marc-145 cells and porcine macrophages, and cGAS-STING pathway possesses an important function against PRRSV infection.

## 4. Discussion

The complex interaction between PRRSV and host immune response remains to be fully understood [32]. Specifically, on the one hand, PSSRV exhibits extensive and intensive suppression of innate immunity, whereas on the other hand, the innate immunity exerts an important role in defense against PRRSV [12,14]. As the RNA virus, PRRSV was thought to be recognized by innate immune receptors TLR3/7/8/9 and RLRs [10]. In our separate study, we systemically dissected the anti-PRRSV activities of nine porcine innate immune signaling adaptors which encompass all known TLR, RLR, NLR, CLR and CDR innate signaling pathways [33,34]. The results from the study suggest that multiple porcine PRR signaling pathways might be involved in the sensing of and fighting against PRRSV, which are more than previously appreciated RNA sensing innate receptors TLRs and RLRs. In this study, by utilizing ectopic expression cells, knockout/knockdown cells, and agonist stimulation, we show that signaling adaptor STING is clearly involved in the anti-PRRSV activity during PRRSV infection. Further, the DNA sensor cGAS agonist was also shown to be anti-PRRSV in both Marc-145 cells and porcine macrophages. Thus, our current results clearly demonstrate that DNA sensing cGAS-STING pathway plays an important role in sensing and controlling PRRSV infection.

Although the role of cGAS-STING in sensing of DNA viruses is clearly elucidated, many RNA viruses of families *Flaviviridae* and *Coronaviridae* have been shown to be restricted by a cGAS and/or STING signaling, and on the other side, the RNA viral proteins can function as antagonists of cGAS-STING pathway, which has been summarized in review [35]. More recent evidence showed that the cGAS-STING pathway is involved in sensing and subjected to evasion of other RNA viruses including murine norovirus (MNV) of family *Caliciviridae* [36], nipah virus (NiV), and measles virus (MeV) of family *Paramyxovirdae* [26], chikungunya virus of family *Togaviridae* [37]. Here, we add one more RNA virus—PRRSV of family *Arteriviridae* to the list.

PRRSV replicates within the cytoplasm using its own RNA-dependent RNA polymerase (nsp9) and its replication cycle does not rely on a DNA intermediate step [4]. While cGAS is exclusively activated upon binding to dsDNA [38,39] or DNA/RNA hybrid [40], one could speculate about the identity of the cGAS agonist during PRRSV infection. In this regard, several sources/possibilities may exist during PRRSV infection. First, PRRSV infection causes mitochondrial damage, inducing cell apoptosis [41,42] and mitochondrial DNA leakage into cytoplasm, where the DNA will be sensed by cGAS, which leads to activation of cGAS-STING pathway. Second, cGAS can be activated by infection induced cell–cell fusion [43]. Additionally, STING can be activated independent of cGAS during virus infection, such as STING activation by cross-talk with RNA sensor RIG-I-MAVS complex [44], by virus-induced necroptosis [45], and by viral envelope mediated fusion process [46]. All these possibilities need to be examined in PRRSV infection.

Since PRRSV infection is sensed and controlled by multiple innate immune signaling pathways, it would raise several questions. First, how much is the relative contribution of these individual signaling pathways in sensing and controlling PRRSV replication during infection? Currently, this is still unknown even though our studies suggested RLR-MAVS is likely the major sensor and antiviral pathway. To address the question, the individual receptors and/or signaling adaptors need to be knockout from cells and then PRRSV replication be examined and compared with each other. Second, what are the interrelationships between these different anti-PRRSV innate immune signaling pathways? Are they separate, cooperative or antagonizing? This information can be obtained by investigation of the PRRSV infection of various knockout cells and the anti-PRRSV activity by different signaling agonists. Such information will be critical for guidance and the design of anti-PRRSV combo therapeutics.

PRRSV functional pathogenesis may vary depending on the PRRSV genotypes. In China, the epidemiological studies including ours showed that highly pathogenic (HP)-PRRSV2 (lineage 8) has been the predominant genotype, whereas the NADC30-like PRRSV2 (lineage 1) isolates increasingly appear in recent years, and QYYZ-like PRRSV2 (lineage 3), CH-1a-like PRRSV2 (lineage 5) and PRRSV1 isolates also coexist in Chinese swine herds [47]. HP-PRRSV is most virulent and is therefore the most important genotype associated with the PRRS and the economic loss caused by PRRS. The PRRSV XJ17-5 strain used in this study, as the representative HP-PRRSV2 (lineage 8) we isolated and well characterized previously [28], should be a relevant genotype. However, we cannot exclude that other genotypes may vary in terms of innate sensing signaling and antiviral pathways, which also need to be further investigated. In summary, our study clearly demonstrates that the DNA sensing cGAS-STING pathway is involved in sensing of PRRSV infection and plays a role in the control of PPRSV infection. However, the viral evasion of cGAS-STING pathway, the relative contribution of this DNA pathway and its relation with other anti-PRRSV innate signaling pathways in sensing and controlling PRRSV infection all need to be evaluated and warrant further study in the future.

## Figures and Tables

**Table 1:** PCR primers used for the construction of PRRSV XJ17-5 infectious clones.

Primer Names	Primer Sequences (5′-3′) *
XJ17-5-*Pac*I-F1 XJ17-5 -*Afl*II-R1 XJ17-5 -*Afl*II-F2 XJ17-5 -*Asc*I-R2 XJ17-5 -*Asc*I-F3 XJ17-5 -1R3 XJ17-5 -*Not*I-2R3 dsRed- *Kpn*I-F dsRed- *Bcl*I-R	AGCTCGTTAATTAATACATGACGTATAGGTGTTGGCT CATAGGTGCTTAAGTTCATTACCACCTGTAACGGAT ATCCGTTACAGGTGGTAATGAACTTAAGCACCTATG CCTTTCTGGCGCGCCCGAAAC GTTTCGGGCGCGCCAGAAAGG ***AGCGAGGAGGCTGGGACCAT**GCCGGCC*TTTTTTTTT TTTTTTTTTTTTAATTACGGCCGCATGGTTCT ACAGGCGGCCGC*GTCCCATTCGCCATTACCGAGGGG* *ACGGTCCCCTCGGAATGTTGCCCAGCCGGCGCC**AGC*** ***GAGGAGGCTGGGACCAT**^#^* ATTGAAGGTACCGCCACCATGGCCTCCTCCGAGGA TGCCGCGGAATGATCACTACAGGAACAGGTGGTGGC

* The restriction enzyme sites used for cloning purposes were underlined. ^#^ The hepatitis D virus ribozyme sequence was shown in italic and the overlapped region was highlighted in bold.

**Table 2:** The CRISPR gRNA encoding DNA sequences and PCR primers for monkey STING genes.

gRNA Names	gRNA Encoding DNA Sequences (5′-3′)
STING gRNA1-F STING gRNA1-R STING gRNA2-F STING gRNA2-R STING gRNA-PCR-F STING gRNA-PCR-R	CACCGTGGATGGATGCAGACTGGAG AAACCTCCAGTCTGCATCCATCCAC CACCGCCATCCATCCCGTGTCCCAG AAACCTGGGACACGGGATGGATGGC PCR Primer Sequences TCGCAGAGACAGGAGCTTTG GGCTGCAGACCCCATTTAAC

**Table 3:** The siRNA sequences for porcine STING genes.

siRNA Name	Primer Sequences (5′-3′)
siSTING-11-F siSTING-11-R siSTING- 529-F siSTING- 529-R siSTING-689-F siSTING-689-R siNC-F siNC-R	CCAGCCUGCAUCCAUCCAUTT AUGGAUGGAUGCAGGCUGGTT GCUCGGAUCCAAGCUUAUATT UAUAAGCUUGGAUCCGAGCTT CCGACCGUGCUGGCAUCAATT UUGAUGCCAGCACGGUCGGTT UUCUCCGAACGUGUCACGUTT ACGUGACACGUUCGGAGAATT

Note: siNC denotes the negative control siRNA.

**Table 4:** Primers for RT-qPCR in this study.

Primer Names	Primer Sequences (5′-3′)
PRRSV Npro-F PRRSV Npro-R S-β-actin-F S-β-actin-R	ATAACAACGGCAAGCAGCAG CTCTGGACTGGTTTTGTTG ATGAAGATCAAGATCATCGCG TCGTACTCCTGCTTGCTGATC

## Data Availability

The data presented in this study are available in the article.

## Supplementary Materials

The following are available online at https://www.mdpi.com/article/10.3390/v13091829/s1, Figure S1: The STING gene insertion and deletion in Marc-145 cell clones from CRISPR gRNA treatment.
