# Supplementary material for: The Innate Immune DNA Sensing cGAS-STING Signaling Pathway Mediates Anti-PRRSV Function
Source: Viruses. 2021 Sep 14;13(9):1829. doi: 10.3390/v13091829 (PMC8473166; doi:10.3390/v13091829)
Supplement: Supplementary file 1 [file viruses-13-01829-s001.zip › viruses-1344706-SI.pdf]

Supplementary

1-4-2

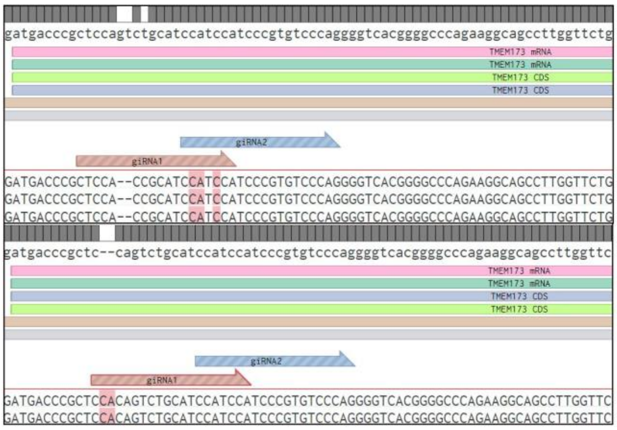

2-7-1

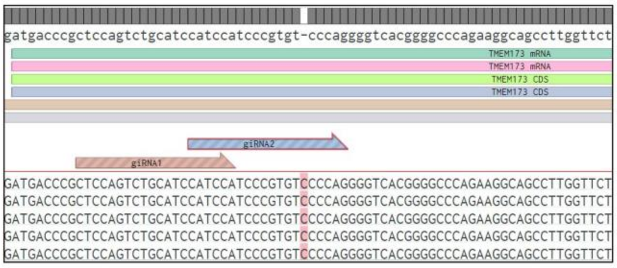

2-7-6

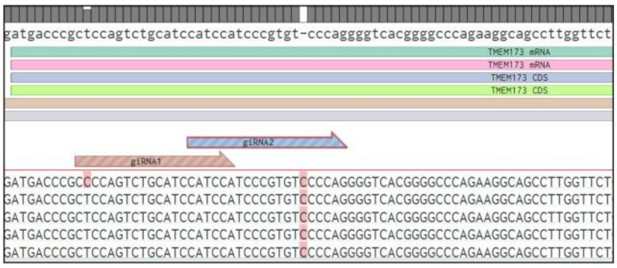

2-7-12

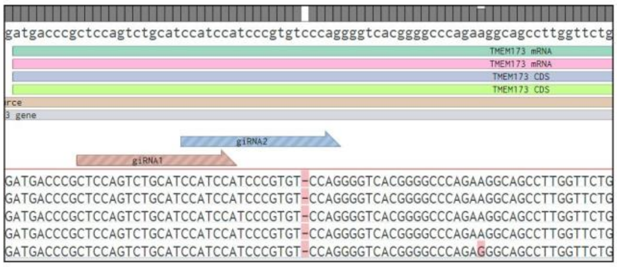

1-4-4

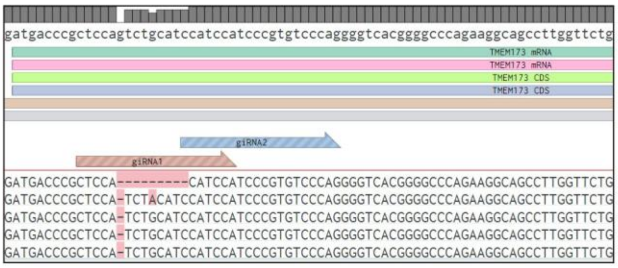

Supplementary Figure S1. The STING gene insertion and deletion in Marc-145 cell clones from CRISPR gRNA treatment.

The monkey STING genome DNA region covering Table 2. the PCR products of individual Marc-145 clones of STING were cloned into T vectors and sequenced for analysis of base insertion/deletion (indel) mutations. As indicated, the four cell clones including 1-4-2, 2-7-1, 2-7-6 and 2-7-12 are STING homologous knockout cells, whereas cell clone 14-4 is likely STING heterologous knockout cells.
